# How Does a Neuron “know” to Modulate Its Epigenetic Machinery in Response to Early-Life Environment/Experience?

**DOI:** 10.3389/fpsyt.2013.00089

**Published:** 2013-08-15

**Authors:** Carley A. Karsten, Tallie Z. Baram

**Affiliations:** ^1^Department of Anatomy and Neurobiology, University of California-Irvine, Irvine, CA, USA; ^2^Department of Pediatrics, University of California-Irvine, Irvine, CA, USA

**Keywords:** synapses, corticotropin-releasing hormone, CRF, glutamate, hypothalamus, epigenetics, stress, maternal care

## Abstract

Exciting information is emerging about epigenetic mechanisms and their role in long-lasting changes of neuronal gene expression. Whereas these mechanisms are active throughout life, recent findings point to a critical window of early postnatal development during which neuronal gene expression may be persistently “re-programed” via epigenetic modifications. However, it remains unclear how the epigenetic machinery is modulated. Here we focus on an important example of early-life programing: the effect of sensory input from the mother on expression patterns of key stress-related genes in the developing brain. We focus on the lasting effects of this early-life experience on corticotropin-releasing hormone (CRH) gene expression in the hypothalamus, and describe recent work that integrates organism-wide signals with cellular signals that in turn impact epigenetic regulation. We describe the operational brain networks that convey sensory input to CRH-expressing cells, and highlight the resulting “re-wiring” of synaptic connectivity to these neurons. We then move from intercellular to intracellular mechanisms, speculating about the induction, and maintenance of lifelong CRH repression provoked by early-life experience. Elucidating such pathways is critical for understanding the enduring links between experience and gene expression. In the context of responses to stress, such mechanisms should contribute to vulnerability or resilience to post-traumatic stress disorder (PTSD) and other stress-related disorders.

## Introduction

Neuronal gene expression is amenable to re-programing by environment and experience (1–3). The neuroendocrine stress axis is influenced by environment and experience during early postnatal development, and these changes endure. For example, maternal-derived sensory input is critical for setting the tone of the hypothalamus-pituitary-adrenal (HPA) axis for life via changes in the expression of glucocorticoid receptor (GR) in the hippocampus and of hypothalamic corticotropin-releasing hormone (CRH). High levels, or predictable bouts, of maternal-derived sensory stimulation result in an attenuated stress response and resilience to stress (4, 5). In contrast, early-life stress causes adults to exhibit augmented stress responses and cognitive impairments, associated with changes in expression of CRH and GR (6–8). Recently, it has been proposed that it is the *patterns* of maternal care that contribute crucially to the perception of stress early in life, and to the subsequent modulation of brain function. Thus, chaotic, fragmented sensory inputs from the mother influence neuronal networks involved in stress for the life of the animal in a direction opposite to that of predictable and consistent patterns (9). Thus, an important common basis may exist for both the beneficial and the adverse consequences of early-life experiences: the pattern of sensory input onto the developing brain might constitute an important parameter that influences the function of stress-sensitive neurons throughout life.

It is suspected that the endurance of the effects of sensory input during this critical period derives from activation of epigenetic mechanisms leading to changes in gene expression that are maintained throughout the lifetime. Here we review the neuroanatomical and molecular pathways bridging sensory input on a whole-brain scale with gene expression programing after distinct early-life experiences. We discuss the implications of these processes to post-traumatic stress disorder (PTSD).

## Epigenetics and Early-Life Experience

The nature of epigenetic mechanisms is amply discussed throughout this collection of papers, and will not be described in detail here. Epigenetics offers an enticing explanation for how relatively brief sensory experiences may lead to long-lasting changes in neuronal function. Indeed, changes in components of chromatin, including DNA methylation or histone modifications have been examined after early-life experience, and found in several key genes involved in regulation of the HPA axis [GR, (10); CRH, (11); arginine vasopressin, (12)]. Here we focus on the lasting repression of CRH in hypothalamic neurons that results from positive maternal care early in life (13). This finding has been confirmed by numerous subsequent studies (4, 5). We focus on the CRH gene both as an important regulator of the stress response (14) and as a likely contributor to the phenotype engendered by nurturing early-life maternal signals, because modulation of CRH function through blocking of CRH receptor type 1 recapitulated the effects of augmented maternal care in non-nurtured pups (15). A second reason for a focus on the CRH gene is its use as a “marker” gene: the reliable detection of CRH repression after augmented maternal care suggests that understanding the mechanism that represses CRH expression enduringly might provide a key to understanding general processes that influence expression programs involving numerous other genes as well. Finally, in the context of the current review, a significant body of literature has implicated aberrant expression and central (CSF) release of CRH in the pathophysiology of PTSD (16–19).

## How Does a CRH-Expressing Neuron Know to Modulate CRH Gene Expression?

**Figure 1:**
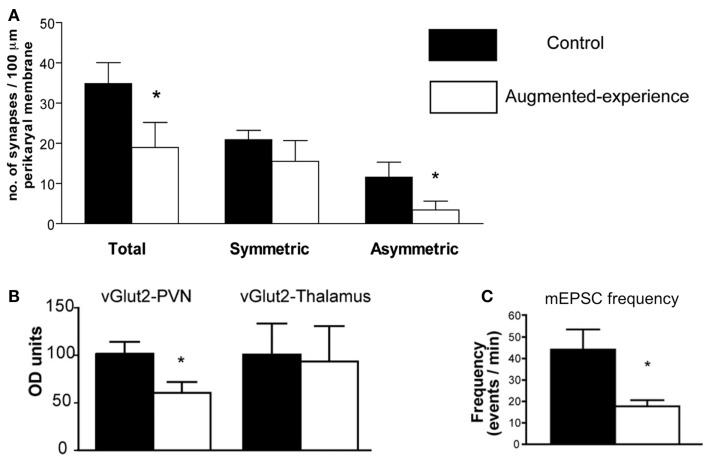
**Augmented early-life experience reduces the number and function of excitatory synapses in the paraventricular nucleus of the hypothalamus (PVN)**. **(A)** Total number of synapses was reduced by 50%, attributable to a 70% reduction of asymmetric (excitatory) synapses onto CRH-expressing neurons in the PVN. **(B)** Levels of the vesicular transporter vGlut2, a marker of glutamate-containing synaptic vesicles, were reduced by approximately 40% in rats with augmented early-life experience relative to controls. **(C)** Miniature excitatory postsynaptic currents (mEPSC) frequency was reduced by 60% in putative CRH neurons. Adapted from Ref. (11) with permission from the Journal of Neuroscience.

Corticotropin-releasing hormone gene expression is regulated by transcription factors, and these in turn are activated by signals that reach the nucleus from the membrane, and often involve calcium signaling (20). Synaptic input onto the CRH-expressing neuron includes a number of neurotransmitters, of which glutamate constitutes a major excitatory input (21). Indeed, glutamatergic signaling in the PVN is necessary for the initiation of the endocrine stress response, and glutamate receptor agonists delivered to the PVN drive CRH release (22, 23). Recent research has revealed that early-life augmented care leads to a transient reduction in the number and function of glutamatergic synapses to CRH neurons in the PVN (11). Using several methods (immunohistochemistry, electron microscopy, and electrophysiology), Korosi et al. discovered that (1) the number of glutamatergic terminals abutting CRH-positive neurons was reduced, (2) the number of asymmetric, putative excitatory terminal boutons onto CRH neurons was reduced, and (3) the frequency of spontaneous excitatory postsynaptic currents to PVN neurons was dramatically reduced (Figure 1). The same measures were taken in the thalamus and yielded no changes. Similarly, there were no changes in markers of inhibitory transmission. Together these data strongly support the notion that augmented maternal care reduces excitatory drive to the CRH-expressing neuron in the PVN.

Whereas the correlation between reduction in excitation and reduction of CRH expression is suggestive, it does not answer the question of causality: is reduced glutamatergic input to a CRH cell required and sufficient to repress CRH? To address this question, *in vitro* methods have been initiated, with the use of organotypic hypothalamic slice cultures to isolate the PVN. In this system, application of glutamate receptor antagonists (blocking both AMPA- and NMDA-type receptors) can effectively eliminate ionotropic glutamatergic transmission. Pilot data suggests that this manipulation may suffice to repress CRH mRNA levels compared to vehicle-treated controls (24). These initial findings are consistent with the notion that augmented maternal care reduces excitatory drive to the PVN, which in turn leads to reduced CRH mRNA production.

## How Does the Sensory Signal from Maternal Care Reach the PVN and Serve to Reduce Excitatory Synapse Number and Function?

Maternal input to her progeny consists of a variety of stimuli, among which sensory stimuli and especially touch (licking, grooming) appear to be the most important (25–27). Levine’s group demonstrated that augmented HPA responses to stress caused by 24 h maternal deprivation could be prevented by stroking the pups, highlighting the importance of tactile stimulation to normal development of HPA activity (28). Using brain-mapping methods, the pathways through which these signals reach the PVN have been identified (29).

**Figure 2:**
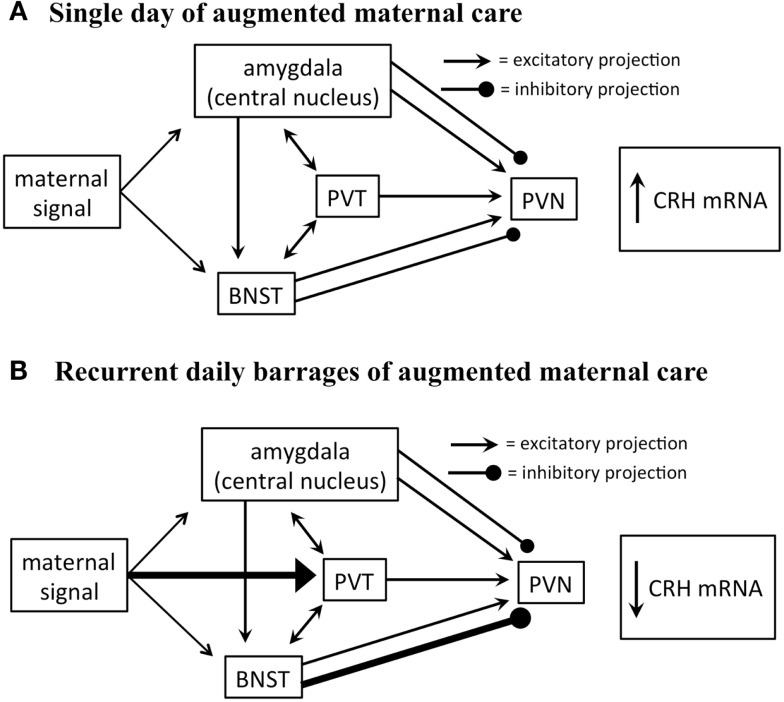
**Proposed circuitry involved in conveying maternal-derived sensory input to CRH-expressing neurons in the PVN**. The PVN receives excitatory and inhibitory projections, including projections from the amygdala, paraventricular thalamic nucleus (PVT), and bed nucleus of the stria terminalis (BNST). These regions are also interconnected by excitatory projections (solid black lines). **(A)** The amygdala and BNST are both activated after a single day of handling-evoked augmented maternal care, and in turn stimulate the PVN (29). **(B)** The PVT is not activated after a single day of augmented maternally derived sensory input, but is recruited by recurrent daily barrages. This is thought to activate regions of the BNST that inhibit CRH-expressing neurons in the PVN (31). It is not fully known how this series of events promotes reduced numbers of excitatory synapses on CRH-expressing neurons.

Glutamate-specific retrograde tracing revealed that excitatory afferents terminating in the PVN originate in the paraventricular thalamus (PVT), lateral septum, bed nucleus of the stria terminalis (BNST), and amygdala (30). The BNST integrates and relays signals from the limbic forebrain and amygdala and provides both inhibitory and excitatory drive to the PVN. Specifically, posterior sub-regions inhibit stress-induced CRH expression in the PVN, whereas anterior regions facilitate it (31). The central nucleus of the amygdala (CeA), important for integration of autonomic inputs, facilitates CRH release from the PVN (Figure 2), likely via the BNST (32, 33).

Importantly, both the CeA and BNST are activated by maternal care. Handling rat pups evokes a burst of nurturing behavior (licking and grooming) by the dam upon the pups’ return to the home cage. A single instance of handling results in c-fos activation in both BNST and CeA (29), yet did not influence CRH expression. In contrast, recurrent handling for a week, which led to repression of CRH expression, was associated with c-fos activation also within the PVT (29). This suggests that the contribution of the PVT to the overall circuit that conveys maternal signals to the CRH cells in the PVN is important to reduce the expression of the gene. The PVT has been shown to play an important role in stress memory and adaptation (34, 35). The PVT sends afferents to the PVN, and possesses bidirectional connections with the CeA and BNST (36). Considering that the majority of PVT output to the structures described above are excitatory, how might PVT activation result in repression of the PVN? Here, we speculate that activation of the PVT might excite BNST regions that are known to inhibit CRH expression in the PVN (Figure 2).

## Initiation vs. Maintenance of Epigenetic Repression of CRH by Early-Life Experience

When considering the changes in gene expression that occur after augmented maternal care, it is important to note two key differences in timing. Repression of CRH begins around postnatal day 9 and persists through adulthood, while changes in glutamatergic signaling to the PVN were noted only at P9 and were back to control levels by P45 (11). This suggests that following the initiation signal mediated by reduction of glutamatergic signaling, there may be additional factors that are involved in maintaining the repression of gene expression that persists long past the initiating signal. Such factors are likely to be epigenetic in nature.

A likely suspect is the neuronal repressor neuron restrictive silencer factor (NRSF). NRSF is a transcription factor that silences gene expression via epigenetic modifications. The CRH intron contains a functional NRSF binding sequence (37), suggesting that the programing of the *crh* gene during early postnatal life may be due to NRSF activity. In fact, NRSF levels in the PVN are dramatically upregulated following augmented maternal care, starting at P9 and persisting into adulthood (11). This pattern is an inverse correlate of CRH expression levels following augmented maternal care, supporting the idea that NRSF may be involved in mediating CRH repression.

## Implications for PTSD

Post-traumatic stress disorder is often associated with a history of early-life trauma (19, 38–40), and more specifically with chronic stressful situations such as abuse and long-lasting war rather than an acute event (41–47). PTSD is characterized by a persistently dysregulated stress response (19, 48, 49), and it is reasonable to assume that chronic early-life stressful events influences an individual’s stress response to promote PTSD. There are several processes that might account for altered stress responses in PTSD. It has been posited that the hypothalamic-pituitary-adrenal axis is permanently sensitized by chronic early-life abuse, and this creates a vulnerability to subsequent trauma, resulting in PTSD. However, the mechanism of such sensitization is unclear. Here we provide a novel and plausible solution: if chronic early-life predictable and nurturing maternal care can reduce excitatory synaptic input onto stress-sensitive neurons in the hypothalamus, and hence “desensitize” future stress responses, then might abusive, erratic, or neglectful maternal behavior provoke the opposite? Augmentation of excitatory input to hypothalamic CRH cells may well serve to sensitize CRH release to future stresses. Whereas this notion is speculative at this point, it is highly amenable to direct testing in animal models. A second possible basis of the abnormal stress response in PTSD that follows early-life chronic stress/abuse may include aberrant regulation of the expression of relevant genes, such as CRH. Here we provide insight into how early-life experience – nurturing or adverse – can result in persistently altered regulation of CRH expression. The lifelong changes in CRH release and expression that result from chronic early-life experiences may provide the neurobiological basis for resilience or vulnerability to subsequent stress, and hence to the development of PTSD.

## Conflict of Interest Statement

The authors declare that the research was conducted in the absence of any commercial or financial relationships that could be construed as a potential conflict of interest.
